# Do gastrointestinal and respiratory signs and symptoms correlate with the severity of gastroesophageal reflux?

**DOI:** 10.1186/1471-230X-12-22

**Published:** 2012-03-21

**Authors:** Hakan Uzun, Demet Alagoz, Mesut Okur, Bunyamin Dikici, Kenan Kocabay, Dursun Ali Senses, Aybars Ozkan, Murat Kaya

**Affiliations:** 1 Department of Pediatrics, Duzce University School of Medicine, 81620 Konuralp, Duzce, Turkey; 2 Department of Pediatric Surgery, Duzce University School of Medicine, Duzce, Turkey

## Abstract

**Background:**

Gastroesophageal reflux (GER) is a disorder that is common by seen in childhood and may lead to severe complications. In this study, we ascertained the incidence of GER among the children who had typical and atypical complaints of GER and whether there was a difference between two groups comparing the findings of 24-hour pH-meter.

**Methods:**

39 out of 70 patients with typical and atypical GER symptoms were diagnosed as GER by 24-hour pH-meter monitoring. The patients were divided into three groups, those having gastrointestinal complaints, those having respiratory complaints and those having both gastrointestinal and respiratory symptoms.

**Results:**

Evaluated the GER prevalence in these groups, it was found to be 60% in the gastrointestinal group, 48.6% in the respiratory group and 75% in the mixed group. When pH-meter measurements of GER positive patients were compared within the clinical groups, the fraction of time that pH was lower than 4 was found to be significantly higher in the mixed group (p = 0.004).

**Conclusions:**

The coexistence of gastrointestinal and respiratory symptoms in the patients with GER may be related to the severe reflux.

## Background

Gastroesophageal reflux (GER) is a condition which develops when reflux of stomach contents causes troublesome symptoms and/or complications [1]. GER can result in typical symptoms such as regurgitation, vomiting, heartburn, abdominal and chest pain and failure to thrive [2]. Extraoesophageal manifestations of GER in children (atypical symptoms) include laryngitis, pharyngitis, sinusitis, otitis media, rinit, and pulmonary symptoms such as asthma, chronic cough and recurrent pulmonary aspiration [3-7]. Twenty-four-hour pH-metry is considered the gold standard test for the diagnosis of GER in children [8].

The aim of this study was to determine the prevalence of GER and to evaluate the twenty-four-hour esophageal pH-metry of pediatric patients who had typical and atypical GER symptoms.

## Methods

### Patient selections

From April 2008 to January 2010, 70 children with suspicion of GER (ages between two-seventeen) complaining of heartburn, abdominal pain, recurrent regurgitation, vomiting, failure to thrive, respiratory symptoms such as recurrent respiratory infection, pharyngitis/tonsilitis, otitis, croup, bronchiolitis, persistent cough, wheezing (non related atopy) seen at the Pediatric Outpatient Clinic, Duzce University Medical School Hospital, Duzce, Turkey, were enrolled in the study prospectively. All patients were questioned by the presence of chronic diseases. Demographic and clinical data including patient age, sex, growth parameters such as length and weight were recorded.

Examined group of 70 patients was divided into 3 subgroups of patients having gastrointestinal complaints, respiratory complaints, and mixed symptoms (gastrointestinal and respiratory symptoms both). The diagnostic method for GER detection was 24 hour esophageal pH-metry

### 24-hour pH metry

The pH metry analysis was performed by an Orion II Ambulatory pH-metry (Medical Measurement Systems Company, Enschede, The Netherlands). The probe was a 2.1-mm outer diameter two sensors placed 5 cm apart, single use pH catheter with a reference electrode (Synectics Medical MMS). Prior to the each test, the electrodes were calibrated in buffer solutions (Reagecon Biomedical, Ireland) at pH 7 and pH 1. This method required placing the pH probe distally until a clearly acidic pH (1.5-2.5) was achieved and then slowly withdrawing the probe until the pH rose to approximately 4.0. At that point, the pH probe was most likely to be in the esophagogastric junction. The probe was then withdrawn to 3-4 cm above the level and fixed at that point. The electrode position was always confirmed by a plain chest radiograph. Before placement of the probe the children had to be fasting for at least 6 hours.

Patients received regular feeds. Daily activities and feeding times were recorded during 24 hours.

Data were analyzed by the MMS investigation and diagnostic software v8.7, for Windows.

All 24 hr pH-metry recordings were manually analyzed and all data underwent visual validation. The DeMeester score was used to define pathological GER [9] recorded by the distal sensor. The following parameters were compared:

1. Number of reflux episodes

2. Number of reflux episodes longer than 5 minutes in 24 hours

3. Longest episode of reflux (in minutes)

4. Fraction of time with pH lower than 4 was considered abnormal if greater than 5%.

5. Reflux index (number of refluxes per hour).

A diagnosis of GER was established when reflux index was greater than >4, or DeMeester score was higher than 14.7, or pathological reflux was considered as at least 1 reflux episode with a pH below 4 in the proximal sensor.

### Statistics

Frequency counts and cross-tabs were used to describe nominal variables. Chi square test was used for the analyses of categorical variables. Man Whitney-*U *test was used for analyses of numeric variables. Kruskal Wallis H was used for analyzing multiple groups and Boferroni adjusted Man Whitney-*U *test was used for multiple comparisons. Statistical analyses were carried out using the SPSS 10.0 for Windows. *P *values < 0.05 were considered significant.

### Ethical considerations

The study protocol was approved by the Ethics Committee of Duzce University Hospital. All subjects gave written informed consent.

## Results

Seventy patients (40 males, 30 females) were enrolled in the study. Demographic Data for the 70 patients was summarized in (Table 1). Frequency of clinical symptoms in patients were such as gastrointestinal 35.7% (n: 25), respiratory 52.9% (n: 37), mixed 11.4% (n: 8) (Table 2). According to the 24 hr pH metry results revealed GER in 39 patients. While GER was detected in 25 patients (64.1%) by the only distal probe, it was detected in 14 patients (35.8%) by the both distal and proximal probes (6 of out 14 patients were included in respiratory group, 5 gastrointestinal group and 3 mixed group).

**Table 1:** Demographic data for the 70 patients included in the study

Variables	GER positive	GER negative	*P*
Age (median)	11 (2-17)	11 (3-17)	0,622^∞^

Male/Female (n)	24/15	16/15	0.204*

Vomiting (n)	8	3	0.255*

Abdominal pain(n)	9	4	0.329*

Nausea (n)	8	9	0.353*

Regurgitation (n)	16	9	0.388*

Hematemesis (n)	1	0	0.207*

Chronic cough (n)	26	21	0.857*

Non atopic Asthma (n)	21	19	0.676*

^∞ ^Man Whitney-U

* Chi-square test

**Table 2:** Distribution of clinical symptom of GER positive and negative patients

	Clinical symptoms			Total
	
n(%)	Gastrointestinal Group 1 n (%)	Respiratory Group 2 n (%)	Mixed Group 3 n(%)	
GER negative	10 (14.3)	19 (27.2)	2 (2.8)	31 (44.3)

GER positive	15 (21.4)	18 (25.7)	6 (8.6)	39 (55.7)

Total	25 (35.7)	37(52.9)	8 (11.4)	70 (100)

The prevalence of GER was found 55.7% among the patients. According to the clinical groups, the prevalence of GER was found to be 60% in the gastrointestinal group, 48.6% in the respiratory group, and 75% in the mixed group.

According to the comparisons of pH meter measurements of GER positive patients in the clinical groups, the in-group median of the fraction of time that pH was lower than 4 were found to be statistically significant. That difference was arisen from the high value in the mixed group. No statistical difference was significantly found between the groups for other parameters (Table 3).

**Table 3:** GER positive pH data by clinical groups

pH metry parameter	Group 1 gastrointestinal median (min max)	Group 2 respiratory median (min- max)	Group 3 mixed median (min-max)	*P*
Number of reflux episodes greater than 5 minutes in 24	2 (1-21)	3 (0-22)	2.5 (2-9)	0.682

Number of reflux episodes in 24 hours	74 (40-426)	61.5 (20-271)	92 (74-197)	0.286

Duration of the longest reflux episode	20 (10-260)	23 (10-146	24 (7-29)	0.919

The reflux index	8 (4-77.8)	7.8 (5-65)	10.3 (7.8-16)	0.576

Fraction of time with pH lower than 4 pH > 5 (%)	2.7 (0-6)	2 (0-10.9)	6 (4.7-11.5)	0.004

The weight and height percentiles of 6 GER positive patients were found to be low. However, no statistically significant difference was found between both groups in comparison with the weight and height percentiles of GER negative and positive patients.

## Dicussion

In our study, 39 of 70 patients (55.7%) who were admitted to hospital with various complaints were diagnosed GER by 24-hour pH monitoring. The distribution of the patients with GER by the initial symptoms at the time of admission was summarized in Table 1, 2.

GER occurs frequently during the first year of life with a peak incidence of 67% at 4 months of age. Also, at least one episode of reflux per day occurs in 50% of infants who are between the ages of birth and 3 months. GER is considered physiologic in early childhood period and disappears in the second year of life. The prevalence of GER was found to be 1.8-8.2% in epidemiological studies among children of 3-17 year of age groups [10]. The frequency of GER was found as 37 to 52.3% in children who were performed by 24-hour esophageal pH-monitoring suspected GER disease [11,12]. In our study GER was found to be 55.7% in patients. High frequency of GER was associated with the severity of the disease.

The frequency of GER was reported to be 25% to 80% among the children with recurring respiratory tract disease in several previous studies, [13]. In our study, the respiratory system symptoms were the most frequently seen finding seen in the patients diagnosed with GER. Respiratory system symptoms were found in 24 cases of whom 18 from respiratory group and 6 from mixed group and it consisted of 60% of 39 patients. The association between GER and respiratory system has been known for a long time. Both esophagus and bronchial tree originated from the same primary nourishing path and were stimulated by the vagus nerve [14]. Khoshoo et al. reported that they found a significant increase in GER incidence in children with asthma. Asthma was found in 50% to 60% of the children with GER [5]. In our study, it was found that 24 out of 39 GER positive patients (61%) had chronic cough and non atopic asthma. Our results were found consistently with the literature.

Several mechanisms have been postulated by which GER might cause coughing. Aspiration of gastric juices containing acid, pepsin, bile acids and duodenal pancreatic enzymes, is considered to be an important mechanism in the etiology of reflux-related cough. Pharyngeal pH recording that demonstrated micro-aspiration of gastric contents into the pharynx favored this hypothesis [15]. In the past, detection of lipid-laden macrophages in bronchoalveolar lavage fluid or sputum has been used as possible a marker for aspiration. Studies show that lipid-laden alveolar macrophages are present in 85% of children with chronic respiratory tract disorders and GER [16,17]. A vagal reflex arc originating from the distal esophagus after either exposure to acid or esophageal distention can cause coughs [18-20]. Acidification of the esophagus can activate local axonal reflexes which can cause inflammation in the airway. A study of Patterson et al. showed that the presence of acid in the esophagus in asthma and chronic cough patients causes releases of tachykinins such as substance P and neurokinin A into the lungs where they cause bronchoconstriction and airway micro vascular leakage [21].

According to the given pH-meter monitoring parameters of GER positive patients in our study, we observed that the parameters were generally high in gastrointestinal and mixed groups. The reasons of high GER positivity in the mixed groups might be that of pH-meter parameters, the number of reflux in 24 hours, the number of reflux prolonged over 5 minutes in 24 hours, and the time interval when esophagus pH was under 4 were high in the mixed group in comparison with other groups. The children in the mixed group, who have both respiratory and gastrointestinal symptoms, are at higher risk of GER. In a study carried out with children population, it was denoted that patients with mixed respiratory and gastrointestinal symptoms had more severe disease than did patients with isolated respiratory disorders indicates that clinical symptoms are a good marker of reflux severity in children [21]. In our study we found similar results.

The limitations of our study are the small number of the patients. The established incidence of GER in examined subgrups of patients is limited only to those with the acidic GER (pH-metry detected). In the remaining examined subjects who did not show the occurrence of acid reflux the presence of alkaline refluks should be suspected. Combined mulitichannel intraluminal impedance and pH measurement should be used. The examined group with mixed GER symptoms is numerically small. The severity of GER should be confirmed by application of diagnostic methods of pH-metry, gastroscopy (eosaphagitis?) and nature of the clinical symptoms observed in the patients. The study should be continued in the future, according to the above remarks, with MI Impedance and pH-metry application.

## Conclusion

24-hour pH-meter is the gold standard non-therapeutic diagnostic method for the diagnosis of GER in children. An important conclusion drawn from the study is that coexistence of gastrointestinal and respiratory symptoms in the patients with GER confirm the severity of reflux.

## Competing interests

The authors declare that they have no competing interests.

## Authors' contributions

HU performed the analysis, writing and preparation of manuscript. DA contributed to data collection. MO contributed to the writing of the manuscript. BD literature review and manuscript preparation. KK substantially contributed to study conception and design, revision of the manuscript. DAS substantially contributed to study conception and design, revision of the manuscript. AO contributed to analysis and interpretation of data, revision of the manuscript, MK contributed to analysis and interpretation of data, revision of the manuscript. All authors read and approved the final manuscript.

## Pre-publication history

The pre-publication history for this paper can be accessed here:

http://www.biomedcentral.com/1471-230X/12/22/prepub
